# Pediatric epididymal schistosomiasis, challenging diagnosis, and implications

**DOI:** 10.1016/j.eucr.2025.102997

**Published:** 2025-03-02

**Authors:** Juliana Arenas Hoyos, Jeff Lawrence Pugach, Lynne M. Eger, Sandy Cope-Yokoyama, Shane F. Batie

**Affiliations:** a Division of Pediatric Urology, Children's Medical Center, University of Texas Southwestern Medical Center, Dallas, TX, USA; b Division of Pediatric Urology, Cook Children's Health Care System, Fort Worth, TX, USA; c Division of Pediatric Infectious Diseases, Cook Children's Health Care System, Fort Worth, TX, 76104, USA; d Division of Pediatric Pathology, Cook Children's Health Care System, TX, USA

## Abstract

Schistosomiasis is a parasitic infection that may be difficult to diagnose in non-endemic regions and may manifest with epididymal involvement.

Testicular schistosomiasis remains a rare disease. The diagnosis is based on clinical suspicion due to a low degree of specificity when it comes to laboratory tests and imaging studies. Treatment mainstays are anthelminthic medication and excision.

We present the first report of epididymal schistosomiasis in a pediatric patient in the United States. Patients require a definitive histologic diagnosis. It is helpful to obtain a thorough history to elucidate exposure to endemic areas as part of assessment and treatment.

## Highlights

• Schistosomiasis (SM) is rare parasitic infection that can present rarely as a pseudotumor of the testes or epididymis.
• Histology is needed for diagnosis.
• Organ sparing surgery may be considered if no involvement is clinically suspected.
• There is scarce information regarding follow-up for systemic sequelae in these cases.

## 1 Introduction

Schistosomiasis (SM) is a parasitic infection that occurs primarily in endemic areas, in communities with inadequate sanitation and limited access to clean water. Genitourinary and bladder SM has been reported in the literature as a risk factor for squamous cell bladder cancer.^1^ As a chronic immune-mediated disease, urogenital SM can result in symptoms such as hematuria, although not all cases exhibit this symptom.

Few cases of epididymal SM have been reported in the pediatric population.^2^ This case is the first reported case of pediatric schistosomiasis involving the epididymis in North America.

## 2 Case presentation

A 10-year-old boy, with no past medical history, was noted by mother to have a right testicular mass. The family denied having noticed the swelling before that time. He was brought to the Emergency department for evaluation 10 days later. He denied pain, fever or urinary tract symptoms. He had no history of hematuria.

On physical examination, there was an enlarged right hemiscrotum and the right epididymis had a hard mass. The right testicle was palpable separate from this mass, slightly larger than left testicle and minimally tender to palpation. There were no skin changes. The left testicle was normal in the scrotum.

**Fig. 1:**
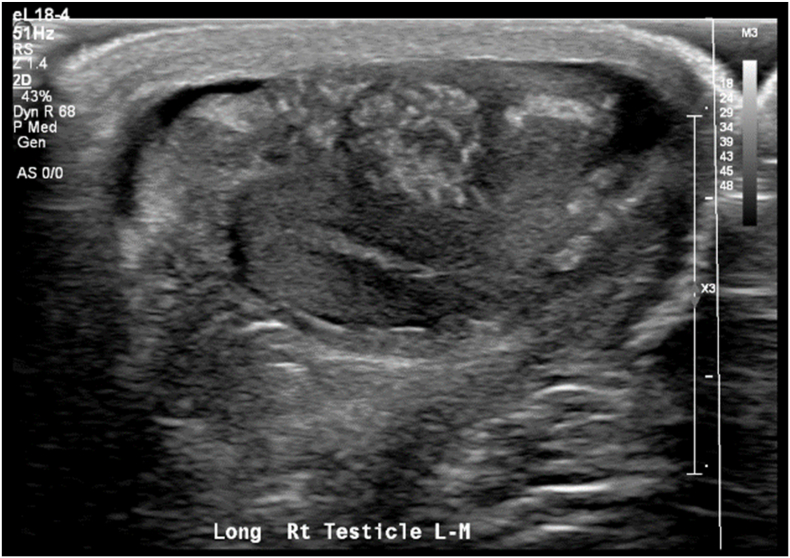
Ultrasound Findings, Epididymal mass with mixed echogenicity and hypervascularity on Doppler.

While in the ED, testicular ultrasound showed a hypervascular right 2.9 x 1.8 × 3.4 cm epididymal mass and mild thickening of the right hemiscrotal wall. (Fig. 1).

Laboratory investigations indicated leukocytosis (Total white blood cell count:17 × 103/ml) with normal eosinophilic count, while urinalysis revealed hematuria (3–6 red blood cells (RBCs)/high-power field) and pyuria (20–25 white blood cells (WBCs)/high-power field). In addition to supportive care, intravenous antibiotics (Cefepime 1 g every 12 hours) were administered. Tumoral serum markers (AFP, BHCG) were obtained and resulted normal, mildly elevated LDH (279 U/L, Normal range 114–237 U/L).

He underwent radical right inguinal orchiectomy. Findings were remarkable for an enlarged firm testicle and epididymis. Scrotal wall was intact. Frozen section was sent, documenting a diffuse granulomatous inflammation of the epididymis, with organisms worrisome for parasite infection.

**Fig. 2:**
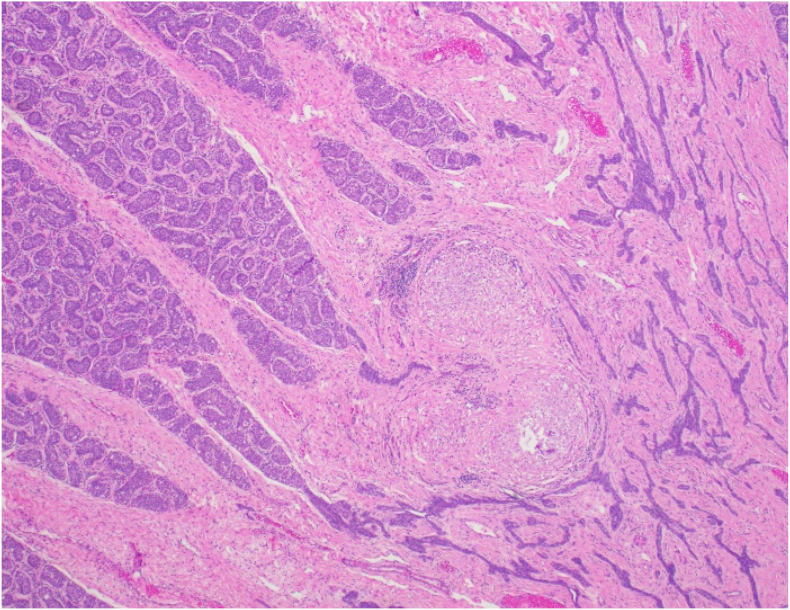
Microscopic examination, Low power view of the seminiferous tubules and rete testis. Granulomatous inflammation and organisms in center, focal involvement of rete testis. H&E, 40x

**Fig. 3:**
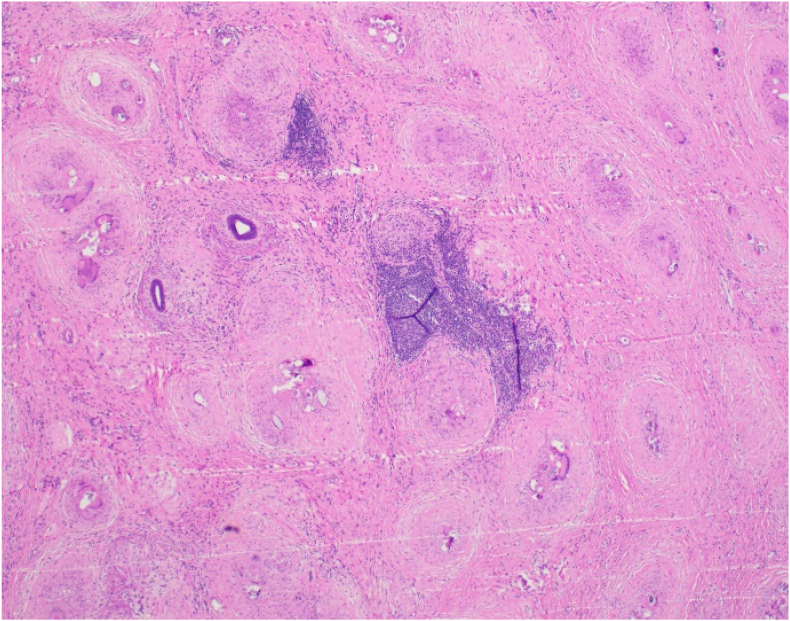
Low power view of epididymis, showing few residual ducts; most are obliterated by granulomatous inflammation and organisms.

**Fig. 4:**
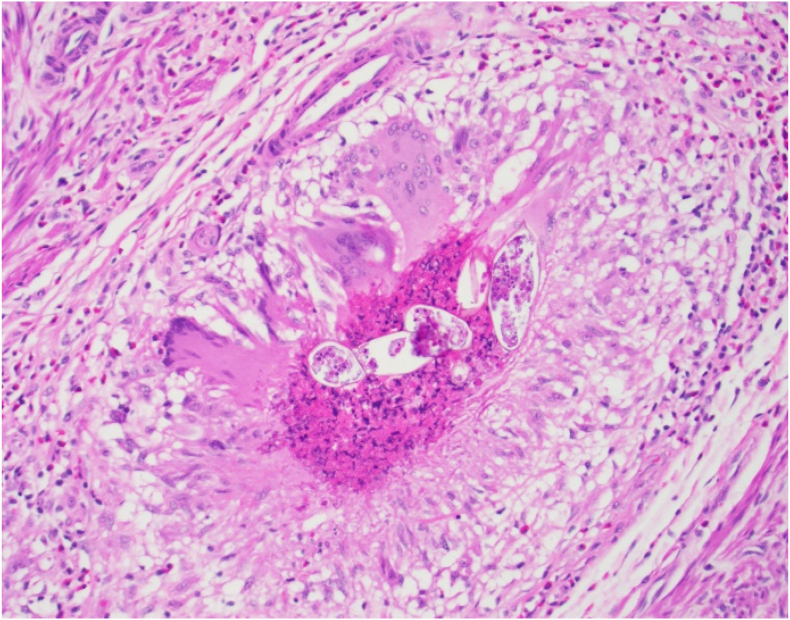
High power view of organisms (including terminal spine), and surrounding granulomatous inflammation. H&E, 200x

The radical orchiectomy specimen showed granulomatous inflammation diffusely involving the epididymis, other excretory ducts, paratesticular fibrovascular tissue, cross sections of the connective tissue of the spermatic cord (including the cord margin section), and focally the rete testis. (Fig. 2). Some of the granulomas contained central necrosis, and there were patchy areas of dense eosinophilic inflammation (Fig. 3). There were abundant parasitic organisms (Eggs/Viable eggs) with morphology consistent with Schistosoma species (Fig. 4). The organisms do not involve seminiferous tubules in the examined sections; the tubules demonstrate typical prepubertal morphology with germ cells present. GMS and AFB special stains are negative. With this pathology there was a confirmed diagnosis of Active Schistosomiasis, likely S. haematobium, and was confined to the specimen.

He was referred to Pediatric Infectious Diseases clinic, history confirmed that family had lived in Sudan for 2 years, while there, the family spent one month in Malawi in 2022. Parents and their four children also visited the tourist area of Salima, and swam in a river adjacent to Salima The nearby Lake Malawi and the rivers in the vicinity are known to harbor Schistosomiasis.

Further testing included negative urine ova and parasite screen, and normal urinalysis with no hematuria. Sonographic evaluation of the kidneys and bladder was performed, with no abnormalities. Schistosoma antibody was positive.

The patient was then prescribed praziquantel 20 mg/kg BID x 1 day (900 mg po BID). Centers for Disease Control and Prevention (CDC) Parasitology was consulted and recommended treatment with praziquantel, and that the other family members all be evaluated, since all swam in Malawi. He was seen for follow up 3 months later and was doing well without extratesticular pathology. His two brothers also had positive Schistosoma antibodies, negative urine O&P, and normal urinalyses. They were also treated with praziquantel. A younger sister had negative screening and was not treated. Parents were encouraged to see their physicians for testing for Schistosomiasis.

## 3 Discussion

The Schistosoma genus contains five species that infect humans, of which S. mansoni and S. haematobium are the most common.^3^ Consensus on the definitions of clinical aspects of schistosomiasis in migrants and travelers, including acute or chronic; possible, probable, or confirmed; active; and complicated schistosomiasis was described in 2024.^3^ Recent reports had described and specified the current endemic areas for Schistosomiasis in Sub-Saharan Africa, and initiatives to eradication.^4^ The World Health Organization reported that 229 million people were affected by schistosomiasis in 2015, with more than 90 % of them living in sub-Saharan Africa.^5^

The urinary bladder and the intestines are the most frequently affected organs. The diagnosis of schistosomiasis is often made by incidental histological findings. Radiological and clinical characteristics resemble the changes caused by tuberculosis in the urinary tract.^6^ As part of the differential diagnoses, prepubertal epididymal masses as epididymal rhabdomyosarcoma should be considered.

Primary schistosomiasis of the testis and epididymis is believed to be caused by the migration of pulmonary larvae into the venous system. As Schistosoma matures in the genitourinary venous plexus, it excretes eggs that cause granulomatous inflammation, resulting in the development of the disease.^7^ The rarity of the primary testicular and epididymal SM is attributed to the narrowness of the veins of the rete testis^8^

The pathologic changes in the urinary tract caused by schistosomiasis are more common in chronic infections than in acute infections. The development of these changes results from the deposition of perivascular eggs, which induces chronic inflammatory lesions, granulomas, and fibrosis as a result.^9^

The prostate and seminal vesicles have been found to have Schistosomal infection at autopsy of male cadavers in endemic regions. Schistosomiasis should be considered when there are calcifications in those organs, and in the setting of chronic prostatitis and vesiculitis, particularly in the appropriate epidemiologic setting.^4^

Schistosomiasis should be considered as a possible cause of acute epididymo-orchitis in patients living in or with recent travel history to endemic areas. There have been few published reports of primary acute testicular or epididymal Schistosomiasis (Table 1). There has been no publication of cases in North America.^2^^,^^10^, ^11^, ^12^, ^13^, ^14^, ^15^, ^16^

**Table 1:** Cases published in the literature.

Author, Year	Country	N	Age at presentation (years)	Symptoms	Assessment	Management
Joshi, 1967^10^	Sierra Leone	1	10	Painless right testicular mass	UA: + SM haematobium eggs	Radical Orchiectomy and treatment for SM
Rambau, 2011^11^	Tanzania	1	9	Left scrotal pain and swelling	UA: (−)	Orchiectomy + Praziquantel
Ekenze, 2015^12^	Nigeria	1	13	Left scrotal swelling and hematuria	UA: + SM haematobium eggs US	Radical orchiectomy + Praziquantel
Alves,2016^13^	Brazil	1	NS	Right scrotal swelling	Negative tumoral markers, US, MRI	Orchiectomy
Oguntunde,2020^14^	Nigeria	1	9	Painless right scrotal swelling	Physical Exam.	Inguinal exploration, biopsy and Praziquantel
Ahmed, 2022^15^	Yemen	1	15	Left testicular swelling	US	Scrotal orchiectomy + Praziquantel.
Ukekwe, 2023^2^	Nigeria	3	7–14	Scrotal swelling	Physical exam, UA (−)	Orchiectomy or biopsy + Praziquantel
Our case	North America	1	8	Progressive right scrotal swelling	UA: (−), Tumoral markers, US	Radical orchiectomy + Praziquantel, Extension to contacts.
Total	–	10	Me: 10, Ave: 10.	–	–	–

A comprehensive review published in 2022,^10^ addressed all the cases to that date of testicular and paratesticular involvement. There was a total of 21 reports for the adult and pediatric population. The median age at presentation was 27 years (IQR 14.5–37.5), however some cases of pediatric presentation are included.

Considering the extent of involvement in most of the cases, including extension to the spermatic cord, inguinal radical orchiectomy is the most suitable approach. Testicular sparing surgery and epididymectomy could be considered, however there are no publications regarding these approaches.

Specific bladder cancer and squamous genitourinary malignancies screening should be considered in these cases. In the absence of hematuria, the evidence regarding screenings squamous bladder cancer, is uncertain.

The presence of a small number of cases in non-endemic areas suggests a need to improve understanding of the presentation and management of this disease, considering the increase in cases due to migration.

## 4 Conclusions

Epididymal schistosomiasis is a rare manifestation of this parasitic disease. We should consider further clinical suspicion in the event of epidemiological contact. In this report, we present a rare presentation of a disease that was considered anecdotic, because of efforts to eradicate contamination of waters and to improve sanitation.

## CRediT authorship contribution statement

**Juliana Arenas Hoyos:** Conceptualization, Data curation, Investigation, Methodology, Project administration, Supervision, Writing – original draft, Writing – review & editing. **Jeff Lawrence Pugach:** Data curation, Formal analysis, Resources, Writing – original draft, Writing – review & editing. **Lynne M. Eger:** Data curation, Writing – review & editing. **Sandy Cope-Yokoyama:** Data curation, Formal analysis, Validation, Visualization, Writing – review & editing. **Shane F. Batie:** Conceptualization, Data curation, Formal analysis, Investigation, Resources, Writing – review & editing.

## Ethical approval

Not required. Informed consent was obtained from all individual participants included in the study.

## Disclosures

None.

## Funding

None.
